# The Role of the Learner in the Cultural Evolution of Vocalizations

**DOI:** 10.3389/fpsyg.2021.667455

**Published:** 2021-08-13

**Authors:** Abby Chopoorian, Yakov Pichkar, Nicole Creanza

**Affiliations:** Department of Biological Sciences, Vanderbilt University, Nashville, TN, United States

**Keywords:** language, cultural evolution, birdsong, learning, culture, computational modeling

## Abstract

As a uniquely human behavior, language is crucial to our understanding of ourselves and of the world around us. Despite centuries of research into how languages have historically developed and how people learn them, fully understanding the origin and evolution of language remains an ongoing challenge. In parallel, researchers have studied the divergence of birdsong in vocal-learning songbirds to uncover broader patterns of cultural evolution. One approach to studying cultural change over time, adapted from biology, focuses on the transmission of socially learned traits, including language, in a population. By studying how learning and the distribution of cultural traits interact at the population level, we can better understand the processes that underlie cultural evolution. Here, we take a two-fold approach to understanding the cultural evolution of vocalizations, with a focus on the role of the learner in cultural transmission. First, we explore previous research on the evolution of social learning, focusing on recent progress regarding the origin and ongoing cultural evolution of both language and birdsong. We then use a spatially explicit population model to investigate the coevolution of culture and learning preferences, with the assumption that selection acts directly on cultural phenotypes and indirectly on learning preferences. Our results suggest that the spatial distribution of learned behaviors can cause unexpected evolutionary patterns of learning. We find that, intuitively, selection for rare cultural phenotypes can indirectly favor a novelty-biased learning strategy. In contrast, selection for common cultural phenotypes leads to cultural homogeneity; we find that there is no selective pressure on learning strategy without cultural variation. Thus, counterintuitively, selection for common cultural traits does not consistently favor conformity bias, and novelty bias can stably persist in this cultural context. We propose that the evolutionary dynamics of learning preferences and cultural biases can depend on the existing variation of learned behaviors, and that this interaction could be important to understanding the origin and evolution of cultural systems such as language and birdsong. Selection acting on learned behaviors may indirectly impose counterintuitive selective pressures on learning strategies, and understanding the cultural landscape is crucial to understanding how patterns of learning might change over time.

## Background: Cultural Evolution and Vocal Learning

For decades, scientists and linguists have studied the evolution of language, searching for the genetic and neural underpinnings of this uniquely human ability as well as exploring changes in languages over time and space (Bateman et al., 1990; Deacon, 1998; Nowak and Krakauer, 1999; Hauser et al., 2002; Kirby et al., 2007, 2008; Croft, 2008; Joseph and Mufwene, 2008; Gray et al., 2009; Fitch, 2010; White, 2010; Bouckaert et al., 2012; Dediu et al., 2013; Mufwene, 2013; Bybee, 2015; Creanza et al., 2015; Tamariz and Kirby, 2016; Blasi et al., 2019; Jarvis, 2019). While numerous genes and neural structures contribute to our ability to learn and produce language, a focus on these bases of the capacity for vocal learning leaves many questions unanswered. Given a large number of individuals to speak with, how do humans decide which features of language to learn, and how do these learning strategies shape the evolution of language? In the songbirds, the largest radiation of vocal learning species, we can similarly ask, how do learning preferences influence the evolution of birdsong and potentially reinforce speciation events in the avian lineage?

Here, we take a two-pronged approach to examine the role of the learner in the cultural evolution of vocalizations. We first briefly survey the literature on the evolution of human language and learned birdsong, focusing on cultural transmission from the perspective of the learner: how do learning predispositions and preferences shape the evolution of learned behaviors? We then propose an agent-based cultural evolutionary model to simulate the relationships between learning preferences and cultural dynamics. We use this simple model to point to challenges and guide the study of vocal learning.

The study of learning is particularly complex because the two evolutionary mechanisms involved, genetic and cultural evolution, occur in parallel and interact with one another (Cavalli-Sforza et al., 1989; Creanza et al., 2015; Creanza and Feldman, 2016). Cultural evolution is defined as change of the form or frequency of socially transmitted behaviors in a population (Cavalli-Sforza and Feldman, 1981; Boyd and Richerson, 1988b; Mesoudi et al., 2006; Creanza et al., 2017). Humans are born with the capacity for language acquisition (Sakai, 2005; Kuhl, 2010, 2011), but any individual language is learned *via* cultural transmission, which is broadly defined as “the attainment of behaviors, attitudes, or technologies through imprinting, conditioning, imitation, active teaching and learning, or combinations of these” (Cavalli-Sforza et al., 1982, p. 19). Most changes in language are products of cultural modifications rather than genetic mutations, with some aspects of language such as sound-level variation in individual vocalizations evolving under neutral cultural evolution (drift), and other aspects such as regularization of verbs and frequency of word-use in a population potentially under cultural selection (Pagel et al., 2007; Creanza et al., 2015; Graham and Fisher, 2015; Newberry et al., 2017; Bentz et al., 2018). While biological evolution is important for explaining the capacity for language at genetic and physiological levels, cultural evolution describes how learned features, such as shifts in language, arise and propagate (Steels, 2011). Since learned traits can alter the selection pressures on genes as well as on other cultural traits, the study of systems such as language must integrate cultural and genetic evolution (Odling-Smee, 1995; Laland et al., 2000, 2001; Rendell et al., 2011; Creanza et al., 2012; John Odling-Smee et al., 2013; Creanza and Feldman, 2014; Fogarty and Creanza, 2017).

When humans learn, they rarely choose at random what to learn or who to learn from; instead, people exhibit learning preferences and cultural biases that may adaptively orient learners towards appropriate tutors and traits (Henrich and Boyd, 1998; Henrich and Gil-White, 2001; Henrich and McElreath, 2003; Efferson et al., 2008; Kendal et al., 2009; Mesoudi, 2011; Acerbi and Bentley, 2014). Children may be more likely to learn something because of its qualities, such as grammatical appeal (Bannard et al., 2013; Culbertson and Newport, 2015) or because others pay attention to it (Tomasello and Farrar, 1986). For example, the tendency of children to track the gaze of others (Emery, 2000) could be paired with information about their social statuses (Tomasello, 1992) to bias learning in favor of certain teachers (Chudek et al., 2012). Grammar and basic vocabulary can be learned by children from their family members (Kerswill, 1996), and this learning is complemented by interacting with peers and other adults (Eckert, 1988). This process begins early, as infants are predisposed towards learning language, such as showing increased attention (Jusczyk and Bertoncini, 1988) or neural activity (January et al., 2009) in response to certain structures and sounds. These learning patterns create frequency-dependent effects, where features important for being understood may be learned through conforming to the majority, whereas innovations to vocabulary and grammar can spread rapidly as unique markers of social groups.

The evolution of language is difficult to study experimentally due to the large amount of linguistic diversity and the length of human generations; birdsong offers an accessible and experimentally tractable analogue that can be used to study the process of vocal learning (Doupe and Kuhl, 1999; Jarvis, 2004; Brenowitz and Beecher, 2005; Brenowitz et al., 2010; Pepperberg, 2011). There are striking similarities between the way that human infants and juvenile songbirds learn how to speak and sing (Bolhuis et al., 2010; Lipkind et al., 2013), including parallels between the social-cognitive mechanisms of vocal learning in humans and songbirds (Fitch, 2009; Fehér, 2017) and between vocal babbling in human children and the plastic subsong of juvenile songbirds (Goldstein et al., 2003; Aronov et al., 2008). Although songbirds and humans are evolutionarily distant, they have similar developmental constraints on vocal learning, which shape cultural diversity (Hyland Bruno et al., 2021). Since both speech and birdsong are culturally transmitted behaviors (Boyd and Richerson, 1988a), the similarities between them can shed light on the process of social transmission. Just as humans bias learning towards potentially adaptive traits, songbirds learning to sing have predispositions and constraints that are crucial to the transmission of their culture, and these “innate predispositions” (Marler, 1990) appear to be genetically determined (Marler, 1970; Marler and Peters, 1977, 2010; Dooling and Searcy, 1980; Soha and Marler, 2000; Hughes et al., 2002; Podos and Warren, 2007).

These predispositions impose limits on cultural diversity by restricting the song features that a juvenile prefers to imitate, and have been observed in many songbird species (Marler, 1990; Lachlan and Feldman, 2003; Shizuka, 2014; Hudson and Shizuka, 2017; Hudson et al., 2020). For example, zebra finch populations exposed solely to atypical songs will learn them, but the features of the learned versions are modified such that they more closely resemble species-stereotypical songs every generation (Fehér et al., 2009). This process is similar to a way in which stable Creole languages have been suggested to form from pidgins: for both spoken and sign languages, when linguistic communication with inconsistent structure is transmitted to the next generation as a first language, the children learning it impart consistent, generalized rules (Singleton and Newport, 2004; Siegel, 2008; Fitch, 2009). Similarly, swamp sparrows exhibit species-specific song selectivity (Dooling and Searcy, 1980) and learn syllables with a conformist bias – a form of biased cultural transmission in which common traits are imitated with a probability that exceeds their frequency in the population (Lachlan et al., 2018).

Conformist bias acts at the level of the individual but has profound effects on the culture of the entire population; in a population that is not highly interconnected, conformity can cause geographically or socially isolated subpopulations to rapidly develop dialects and stable traditions. These dialects have been suggested to play a role in sexual selection. One proposal is that dialects signal that males have survived in and are adapted to the local environment (Marler and Tamura, 1962; Nottebohm, 1969; Podos and Warren, 2007). Such preferences might limit inter-species hybridization (Ortiz-Barrientos et al., 2009), but empirical studies have only occasionally found genetic barriers coincident with dialect boundaries (Nottebohm and Selander, 1972; Baker, 1975; Handford and Nottebohm, 1976; Zink and Barrowclough, 1984; Hafner and Petersen, 1985; Lougheed et al., 1993; MacDougall-Shackleton and MacDougall-Shackleton, 2001; Soha, 2004; Lipshutz et al., 2017; Poesel et al., 2017). Alternatively, birds may prefer to learn local songs because these songs have performed best in local male–male competition (Kroodsma and Miller, 1996; Podos and Warren, 2007). Adaptation to both the abiotic and social environment can lead to dialect formation, which is supported by a correlation between song sharing among neighbors and mating success (Payne et al., 1988; Beecher et al., 2000).

In contrast to bird species with strong innate predispositions for what to learn, other bird species such as the American robin and the gray catbird invent much of their song every generation, in addition to learning certain song elements from conspecifics (Kroodsma et al., 1997; Johnson, 2006). This innovation generates novel syllables in the population, and may exist alongside novelty-biased cultural transmission – the preferential imitation of rarely occurring sounds. Such biases can produce intriguing learning patterns such as those found among heterospecific vocal mimics – songbird species that integrate vocalizations of other species into their repertoires (Rundstrom and Creanza, 2021). Heterospecific vocal mimicry has been hypothesized to evolve due to sexual selection as the result of increased size or complexity of the song repertoire (Hindmarsh, 1986; Jarvis, 2006; Goller and Shizuka, 2018). In this case, the song-learning template might evolve to be less selective, allowing birds to increase their repertoire size by imitating other species in addition to their own (Goller and Shizuka, 2018).

Individual innovation, transmission of culture, and the dynamics of social networks characterize the evolution of culture (Dor and Jablonka, 2001; Tchernichovski et al., 2017). Similarly to birdsong, language changes due to processes of learning and innovation that produce shared linguistic markers and distinct dialects (Fehér, 2017). Just as avian culture may allow for communication and identification of kin (Nowicki and Searcy, 2014), human dialects and languages may develop to favor effective communication and to aid the identification of outsiders (Shutts et al., 2009; Fehér, 2017). The interaction of these processes of innovation, transmission, and group dynamics can describe how learning operates, but not its origin and maintenance. Multiple hypotheses exist to describe the repeated evolution and persistence of vocal learning, focusing on the advantages learners gain from adapting to local environments, communicating with conspecifics or kin, or signaling fitness to mates (Nowicki and Searcy, 2014). However, these existing hypotheses seldom consider how pre-existing vocal variation might influence the evolution of learning. Given the same learning preferences, the evolutionary dynamics of vocalizations might greatly differ in a population with extensive vocal variation versus one with a single established dialect.

Much theoretical and empirical research has focused on the evolution of cultural traits, including their dynamics over time and their interactions with one another (Cavalli-Sforza and Feldman, 1981; Boyd and Richerson, 1988b; Henrich, 2004; Creanza et al., 2017). In parallel, researchers have studied the role of biased transmission, or learning preferences, in cultural evolution (Henrich and Boyd, 1998; Henrich and Gil-White, 2001; Henrich and McElreath, 2003; Efferson et al., 2008; Kendal et al., 2009; Mesoudi, 2011; Acerbi and Bentley, 2014). These two evolutionary forces interact with one another; in particular, cultural transmission biases likely influence the dynamics and spread of cultural traits (Lachlan and Feldman, 2003). A challenge to any hypothesis about the evolution of vocal learning involves how learning is favorable in the context of existing behavioral variation (Nowicki and Searcy, 2014). To illustrate how interactions between unlearned predispositions and learned behaviors can be counterintuitive, we developed and will briefly explore a spatially explicit model. This model only includes several important dynamics, but combines them to illustrate the counterintuitive evolution of learning preferences that occur when selection acts on culture. We then discuss how these results can guide future research of vocal learning.

## Modeling the Role of the Learner in the Cultural Evolution of Vocalizations

### Model Methods

To study the evolution of learning preferences with a focus on the adaptive pressures acting on social learning, we developed a spatially explicit model of cultural evolution. To understand these pressures, we need to determine how learners are influenced by their social environment, to what information they are attuned, and how they progress from observation to production of cultural traits. Whereas others have looked at how learning affects the cultural environment, we focus on how learning preferences are affected by evolutionary processes, including the indirect selection on learning preferences that occurs in concert with selection on learned traits themselves, and we consider the implications of these interactions for the evolution of both language and birdsong.

To better understand the evolution of both learning predispositions and the traits that are learned according to these predispositions, we designed a basic model of how learning may change over time in a population. To model an environment with indirect selection on learning preferences, we constructed a spatial population model that could represent any species with social learning and heritable learning preferences, including humans and songbirds. The model was written in Python 3.8.5 with the following packages: NumPy, SciPy, and Pandas (McKinney, 2010; Harris et al., 2020; Virtanen et al., 2020). Plotting was done using Matplotlib (Hunter, 2007). See https://github.com/CreanzaLab/role_of_the_learner for code and details.

In this model, a square array containing 128 individuals per side is initialized (a total of 16,384 individuals; this is smaller than some populations, but is within reasonable limits of computational time). Each individual is initially assigned one of 16 cultural types at random, as well as a preference for either conformity or novelty. Cultural types can be envisioned as representing different vocalizations: different songs or syllables in a bird population, or different pronunciations or other linguistic features in a human population. Cultural mutations will increase the number of these syllable types over time, whereas selection and drift will decrease this number. Learning preferences are transmitted vertically from parent to offspring, and these preferences cause juveniles to be more likely to learn cultural types that are either more or less common among their neighbors: with a conformity-biased learning preference, learners are disproportionately likely to learn the most common cultural type among their neighbors, and with a novelty-biased learning preference, learners are more likely to learn the rarest cultural type. In the simulations described in detail here, one quarter of individuals prefer novelty, and three quarters prefer conformity at the beginning of each simulation. We also tested a range of initial novelty-conformity proportions to consider how these affected selection. This proportion changed over the course of the simulations, and 25% initial novelty was chosen because it illustrated ongoing selection in some simulations and the lack of selection in others.

Each of the 4,000 timesteps contains three events: mortality, replacement, and learning. During the first event, individuals have a 20% chance of dying, causing their position in the population matrix to become empty. In preliminary tests, we observed that the probability of death accelerated or decelerated, but did not qualitatively change, the trajectory of the simulations. Songbirds have previously been modeled with annual mortality rates as high as 40% (Slater, 1986; Lachlan et al., 2018), reflecting death-rate estimates in wild bird populations (Lack, 1954; Summers-Smith, 1956). However, we also note that this death rate can represent individuals leaving the population for other reasons.) During replacement, these empty locations are filled by juveniles that are the offspring of one of the adjacent individuals in the matrix, such that successful neighbors are more likely to produce offspring. For every location into which a juvenile is born, the living neighbors are surveyed, and those neighbors with cultural types closer in frequency to an “ideal neighbor proportion” (from 0 to 100%) are more likely to be selected to be a parent. In other words, if the ideal neighbor proportion is close to 0%, an individual with a rare cultural type is more likely to leave an offspring; if the ideal neighbor proportion is close to 100%, an individual with a common cultural type is more likely to leave an offspring (for additional details, see code on GitHub). For ideal neighbor proportions of 10 and 90%, we also ran simulations at several initial proportions of novelty-biased learners. The juvenile inherits a learning preference identical to that of the selected neighbor (with a 0.01% chance of being assigned a random learning preference). These mutations of learning strategy exist to balance the random effects that take place at the beginning of simulations, and ensures a standing level of variation in learning strategy. A higher rate of learning mutations would cause the novelty-conformity equilibrium established by selection to tend towards 50%, and would decrease the effects of selection. During the learning step, juveniles choose one of the cultural types to inherit by surveying their neighbors, such that juveniles tend to learn less common cultural types if they have a novelty preference and more common types if they have a conformity preference. All juveniles have a 0.5% chance of inventing a new cultural type, in which case they do not learn another one from a neighbor. The invention of new cultural types can increase rates of selection; they allow for existing novelty-seeking learners to acquire unique syllables, which can affect selection based on the population’s ideal neighbor proportion. Thus, in each timestep, there are two interacting cultural forces affecting cultural evolution: (1) individuals are more likely to reproduce if their cultural trait is at a certain frequency in the local neighborhood, and (2) individuals are more likely to learn cultural traits with certain frequencies based on their learning preferences. As a result, selection is dependent on the cultural type and its frequency, and operates indirectly on learning preference.

In addition to the proportion of novelty or conformity preference in the population, we recorded a measure of cultural patchiness in the population to assess whether individuals with the same cultural type were clustered together. This was calculated by identifying boundaries of each cultural type on the cultural landscape *via* a Sobel operator, which acts as a derivative (individuals surrounded by more unique cultural traits will have a higher derivative). The magnitude of this derivative is calculated for each culture type. Then, we calculate the sum of these magnitudes for all culture types and for the entire population. This sum represents the heterogeneity of the cultural landscape, such that environments with greater cultural diversity, and therefore more boundaries between culture types, have a higher spatial derivative. We use this measure to summarize the trends of cultural homogeneity and compare these trends across simulations.

### Model Results

**Figure 1:**
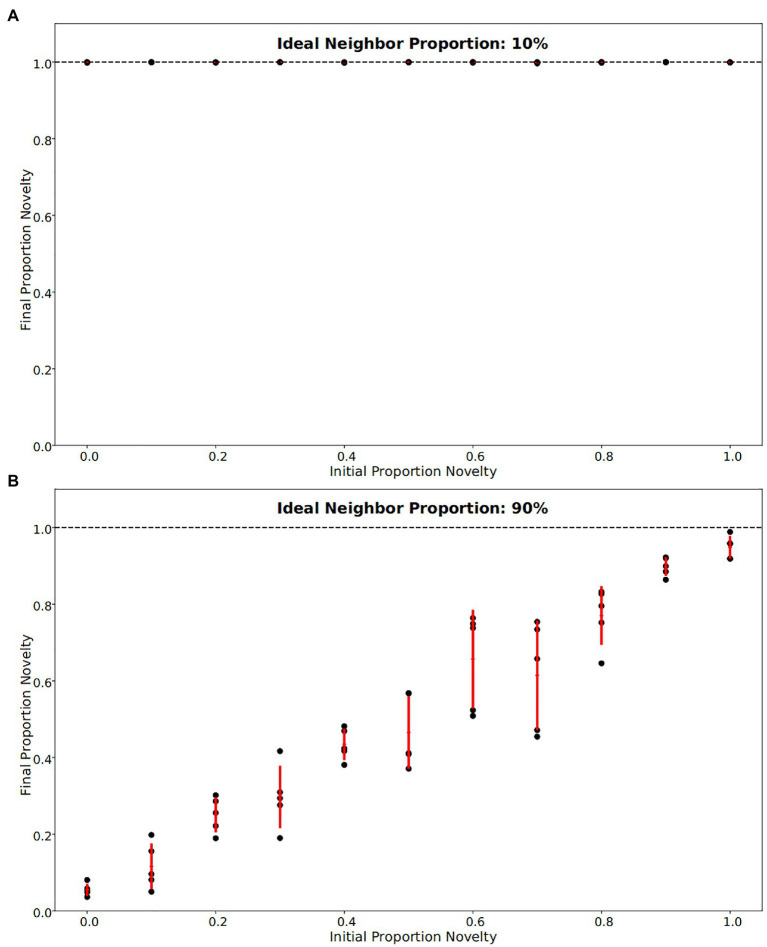
Final fraction of novelty preference in the population after 4,000 iterations, for a range of initial novelty preferences. Each of five replicates per ideal neighbor proportion are represented by a black point, with one standard deviation from the mean marked by red lines. **(A)** For populations in which rare cultural traits are favored (an ideal neighbor proportion of 10%), novelty preference was strongly selected for, such that all populations were almost entirely made up of individuals with a novelty preference at the end of the simulations. **(B)** Populations that favor conformity (ideal neighbor proportion of 90%) show little selection against individuals with novelty preferences, as these remain at nearly the same frequency over the course of the simulations.

**Figure 2:**
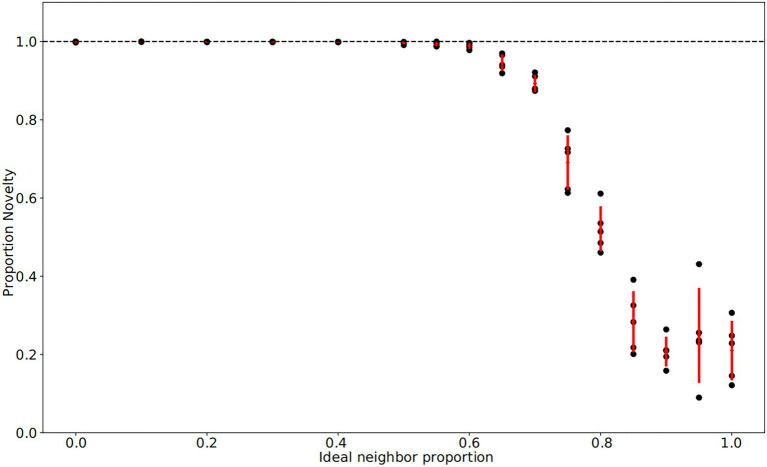
Final fraction of novelty preference in the population after 4,000 iterations. Each population has a particular ideal neighbor proportion, representing the frequency of a cultural trait at which individuals have the highest fitness. As in Figure 1, each of five replicates per ideal neighbor proportion are represented by a black point, with one standard deviation from the mean marked by red lines. Among populations with preferences for cultural conformity (ideal neighbor proportions between 0.85 and 1), the final proportion of novelty-biased learners does not deviate from its initial value of 0.25, suggesting little selective pressure. In contrast, populations with an ideal neighbor proportion below 0.5 exhibit strong selection pressure favoring novelty-biased learners, and this learning preference is nearly ubiquitous at the end of these simulations.

**Figure 3:**
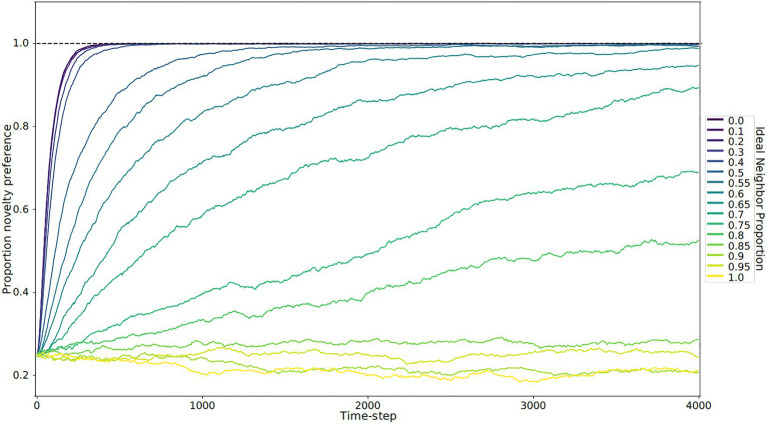
Novelty preference over time by ideal neighbor proportion. Each line represents the average of five replicates. When individuals with a rare cultural type are more likely to leave offspring (low ideal neighbor proportion), there is strong selection for novelty preference (blue lines). Populations with a high ideal neighbor proportion experience little selection for or against novelty preference (yellow and light green lines).

**Figure 4:**
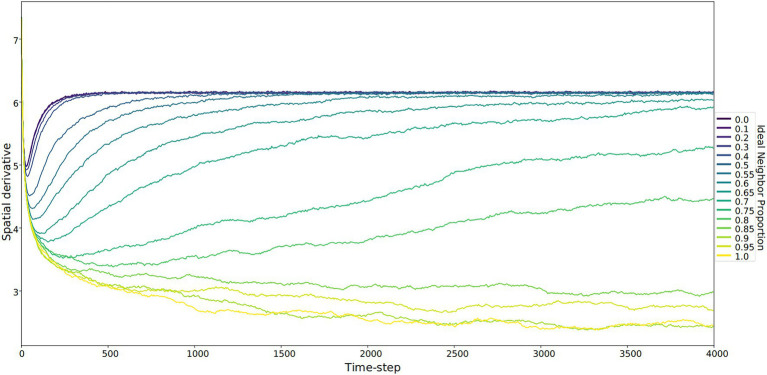
The spatial derivative – calculated as the sum of the Sobel operator, which identifies boundaries between culture types – over time. This sum represents the unevenness of the cultural landscape, averaged for five runs at each ideal neighbor proportion. The larger this value is, the more heterogeneous the landscape. These larger values correspond to lower ideal neighbor proportions (blue lines) – populations in which individuals with uncommon culture types have greater reproductive success. Lower spatial derivative values indicate more homogeneous cultural landscapes, including larger regions with only one culture type. Within these regions, all learners will either learn the only available type or rarely invent a new culture type.

**Figure 5:**
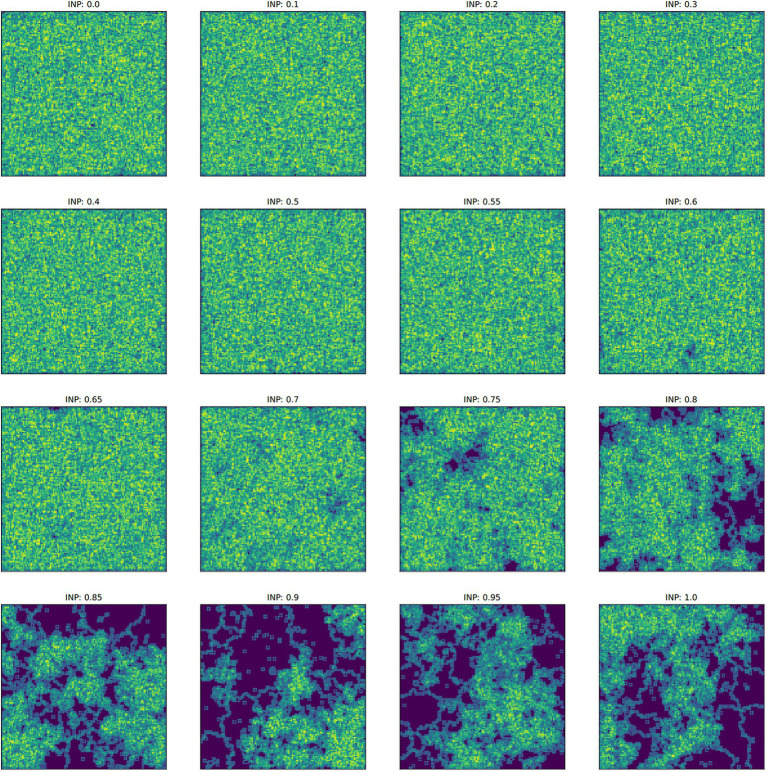
Examples of the final culture matrix for each ideal neighbor proportion (INP) after 4,000 iterations. The Sobel operator produces greater values in regions of higher cultural diversity (yellow and light green) and lower values in regions of homogeneous culture (dark blue). Note that for higher INP, cultural conformity is selected for, but individuals with novelty preference can exist in relatively large numbers, depending on their starting frequency (Figure 1B).

In our simulations of populations with low ideal neighbor proportion – in which cultural uniqueness is favored by selection and thus individuals with rare cultural types are more likely to leave an offspring – novelty seekers became predominant (Figures 1A, 2, 3). As expected, these populations maintained a higher rate of cultural diversity and tended to select strongly against conformist-seeking learners (Figures 4, 5). Further, when cultural uniqueness was favored, novelty seekers predominated in the population at the end of the simulation regardless of how common they were in the initial population (Figure 1A). In contrast, when common cultural traits were favored, the proportion of novelty-seeking learners remained relatively constant over the course of the simulations (Figure 1B). Since we observed this predictable pattern – that the starting proportion of novelty-seeking individuals increases to approach fixation when rare traits are favored and remains relatively constant when common traits are favored – we ran subsequent simulations with 25% of individuals exhibiting the novelty-seeking learning preference and 75% of individuals exhibiting the conformity-biased learning preference.

In populations with low ideal neighbor proportions, novelty seekers predominate over time, whereas in populations with high ideal neighbor proportions, novelty seekers could not be effectively selected against during the simulations (Figure 1). In simulations with a high ideal neighbor proportion and thus a fitness benefit for individuals with a common cultural phenotype, the cultural landscape became dominated by homogeneous cultural regions (Figure 5), in which novelty-seeking learners did not have neighbors with uncommon cultures, and thus could not be selected against. Higher proportions of conformist learners corresponded to more stable landscapes, where large regions contained only a single cultural type (Figures 4, 5). Conversely, populations in which novelty preferences predominated were more heterogeneous. All populations began in an uneven, randomized state since the initial cultural types were evenly spread (Figure 4). Populations with higher ideal neighbor proportions formed more even cultural landscapes, whereas populations with many novelty-seekers remained more uneven (Figure 4). These homogeneous cultural regions spread over time (Figure 4) and represent cultural niches in which there is no disadvantage to an intuitively deleterious trait: in this case, a predisposition towards novelty in populations undergoing selection for cultural conformity (Laland et al., 2000).

## Discussion

Our model focuses on the co-evolution of culture and learning preference, specifically on the evolution of learning preferences due to selection on learned behaviors. Cultural selection on the basis of particular cultural traits (or traits at a certain frequency) could favor particular learning preferences, influencing their frequency in subsequent generations. In our model, selection favoring individuals with uncommon cultural features led to high rates of novelty-biased learners, but selection favoring local conformity did not necessarily increase the frequency of conformity bias in the population. This counterintuitive outcome occurs because a sufficient number of conformists will create culturally homogeneous niches, in which selection against those with a preference for novelty cannot take place, as individuals do not have the opportunity to learn uncommon cultures from their neighbors. Understanding the dynamics that take place between the cultural environment and the learners is particularly relevant for songbirds, whose reproductive success is directly linked to singing a song that appeals to females and repels other males. However, humans and any other species with variable, heritable learning strategies and with selection based on learned traits will experience similar dynamics.

There is evidence that selection on learned behaviors can result in highly adaptive learning processes, such as the development of unlearned predispositions that guide effective cultural transmission. For example, in golden-crowned sparrows, juvenile birds can selectively respond to conspecific songs before the song-learning process begins (Shizuka, 2014; Hudson and Shizuka, 2017); in zebra finches, artificial songs transmitted over several generations become more species-stereotypical (Fehér et al., 2009); in humans, deaf children without access to language have spontaneously produced sign languages by communicating with one another (Goldin-Meadow and Mylander, 1998). This tendency of learning to guide individuals towards useful, species-specific behaviors points to the evolutionary pressures responsible for this adaptation. Notably, our model only considers the transmission of a single cultural trait, taken to represent the phenotype of a vocalization, whereas human and animal cultures often contain many learned behaviors. In addition, we focus on modeling the learning preferences of the learner, although in some cases the phenotype of the tutor can also predict how a trait will be learned. For example, zebra finch pupils improvise more when their tutor has a song with a lower diversity of syllables; this “balanced imitation” strategy can maintain rare song elements and prevent homogenization of songs in a population (Tchernichovski et al., 2021). Since each adult male zebra finch has multiple syllables in his repertoire, an imitation strategy that differs based on the tutor’s cultural repertoire may maintain local cultural diversity in zebra finch populations (Tchernichovski et al., 2021). For individual syllables in the song repertoire, this proposed pattern is similar to our simulations with a medium or low ideal-neighbor preference: the populations maintain a high cultural diversity despite completely new cultural variants being uncommon. High levels of cultural variation in a population could thus be maintained not only when individuals with rare traits have higher fitness, as we show here, but also if attempts to learn certain cultural types result in a higher mutation rate.

When considering the evolution of a cultural system such as language or birdsong, it is important to consider how the learning process originated and how predispositions and preferences guide learning (Lachlan and Slater, 1999; Lachlan and Feldman, 2003). Our model suggests that cultural variation – the presence of diverse heritable behaviors – is necessary for the evolution and maintenance of learning, since the set of existing behaviors places limits on imitative learning. The interaction between a species’ social learning and their access to behaviors to imitate may help explain the learning capacities of bird species (Creanza et al., 2016; Robinson et al., 2019), and may be a driver of human evolution (Rendell et al., 2011). In humans, for example, researchers have observed that different groups prefer to learn in different ways (Mesoudi et al., 2015), and this evolutionary approach suggests that these learning differences are the result of cultural selection favoring different traits. The study of cultural complexes such as language, therefore, should consider the selective pressures imposed on the learning systems themselves. Genetic selection based on learned behavior may contribute to speciation in birds, where preference for more similar dialects could act as a barrier to gene flow (Ortiz-Barrientos et al., 2009). Strategies such as overimitation – in which children imitate unnecessary or irrelevant actions accompanying important ones (Lyons et al., 2007) – are important for their role in individual learning, but also for their exaggeration of existing behavioral diversity, providing variation for selection. Error-prone learning produces behavioral variation that may provide a bet-hedging advantage during selection and increased rates of evolution, whereas error-free learning with conformist preferences may make learning costlier than instinctual behavior (Dor and Jablonka, 2001).

Our model helps expand the existing questions about the evolution of language. Prior to the evolution of a learning strategy optimized for language, we suggest that our ancestors existed in a cultural niche in which related behaviors existed. Researchers have considered the origin of language learning by exploring the interface between behavioral diversity and genetic predispositions to learning (Tomasello, 2009; Richerson et al., 2010; Chudek and Henrich, 2011; O’Brien et al., 2012; Creanza and Feldman, 2016). Some have suggested that cognitive parallels between language and tool-making (Lotem et al., 2017) or foraging (Kolodny et al., 2015) provide possible origins for language-oriented learning. We hypothesize that rates of error in imitating behavior – and the genetic or cultural social norms concerning these behaviors – could have evolved to balance the precision of learning with behavioral plasticity.

Why do songbirds continue to learn songs, despite many other bird species successfully surviving and reproducing without song learning? There are numerous hypotheses about the fitness consequences of song learning (Nottebohm, 1972; Slater, 1989; Lachlan and Slater, 1999; Lachlan and Servedio, 2004; Podos and Warren, 2007; Nowicki and Searcy, 2014); based on our spatial model, we suggest that the best place to conduct research could be the edges of dialect boundaries or subspecies ranges. These are the regions in which the action of evolutionary pressures on learning may be most pronounced, since the higher cultural diversity at such boundaries can reveal inherent preferences in song learning. Behavioral ecologists may be more likely to identify the fitness consequences of song learning at these boundaries, and in addition to field data, can use expanding sources of citizen science data to supplement the discovery of these dialect boundaries in common species (Searfoss et al., 2020).

To better understand the role of the learner in cultural evolution, we propose a simple model of how individuals interact and learn within their social and cultural environments. The results of our simulations suggest that the evolution of learning is driven most strongly by the selection taking place at the level of cultural phenotypes, and that the fitness consequences of this selection are most significant in regions with high cultural diversity. Future evolutionary studies of song and language learning could usefully integrate research from archaeology, anthropology, ecology, and genetics, among others, to uncover the qualities of learned behaviors on which selection occurs.

## Data Availability Statement

The datasets and code presented in this study can be found in online repositories. The names of the repository/repositories and accession number(s) can be found at: https://github.com/CreanzaLab/role_of_the_learner.

## Author Contributions

AC, YP, and NC conceived and designed the study and wrote and edited the manuscript. YP ran simulations and analyzed the data. All authors contributed to the article and approved the submitted version.

## Conflict of Interest

The authors declare that the research was conducted in the absence of any commercial or financial relationships that could be construed as a potential conflict of interest.

## Publisher’s Note

All claims expressed in this article are solely those of the authors and do not necessarily represent those of their affiliated organizations, or those of the publisher, the editors and the reviewers. Any product that may be evaluated in this article, or claim that may be made by its manufacturer, is not guaranteed or endorsed by the publisher.
